# Bison: bisulfite alignment on nodes of a cluster

**DOI:** 10.1186/1471-2105-15-337

**Published:** 2014-10-18

**Authors:** Devon Patrick Ryan, Dan Ehninger

**Affiliations:** German Center for Neurodegenerative Diseases (DZNE), Ludwig-Erhard-Allee 2, Bonn, 53175 Germany

**Keywords:** DNA Methylation, Alignment, Bisulfite sequencing, Computer cluster

## Abstract

**Background:**

DNA methylation changes are associated with a wide array of biological processes. Bisulfite conversion of DNA followed by high-throughput sequencing is increasingly being used to assess genome-wide methylation at single-base resolution. The relative slowness of most commonly used aligners for processing such data introduces an unnecessarily long delay between receipt of raw data and statistical analysis. While this process can be sped-up by using computer clusters, current tools are not designed with them in mind and end-users must create such implementations themselves.

**Results:**

Here, we present a novel BS-seq aligner, Bison, which exploits multiple nodes of a computer cluster to speed up this process and also has increased accuracy. Bison is accompanied by a variety of helper programs and scripts to ease, as much as possible, the process of quality control and preparing results for statistical analysis by a variety of popular R packages. Bison is also accompanied by bison_herd, a variant of Bison with the same output but that can scale to a semi-arbitrary number of nodes, with concomitant increased demands on the underlying message passing interface implementation.

**Conclusions:**

Bison is a new bisulfite-converted short-read aligner providing end users easier scalability for performance gains, more accurate alignments, and a convenient pathway for quality controlling alignments and converting methylation calls into a form appropriate for statistical analysis. Bison and the more scalable bison_herd are natively able to utilize multiple nodes of a computer cluster simultaneously and serve to simplify to the process of creating analysis pipelines.

## Background

DNA methylation involves the covalent modification of cytosine bases and serves to epigenetically regulate a large variety of biological properties, from imprinting to development and its dysregulation is associated with diseases such as cancer [1]. While there are a number of methods currently available to probe DNA methylation (e.g. MeDIP-seq and MRE-seq), the gold-standard for single-base resolution is bisulfite sequencing, sometimes referred to as BS-seq. Here, DNA is treated with bisulfite, which results in the conversion of unmethylated Cytosine (C) to Uracil (read by the sequencer as Thymine (T)), leaving methylated Cs unconverted.

Mapping of bisulfite-converted short reads, such as those commonly produced by the Illumina HiSeq, creates a number of difficulties. Firstly, if both Cs and Ts in reads can map to Cs in the genome, then methylation metrics will be systematically skewed, as methylated reads will map preferentially, given that they contain more information. The alternative is to *in silico* convert both the reads and the genome prior to alignment, thereby producing unbiased, but less efficiently mapped, alignments. This creates a second difficulty in that the two DNA strands are no longer complementary, requiring them to be treated separately. Finally, PCR amplification during library preparation results in the creation of an additional two DNA strands, complementary to the original bisulfite converted strands. The presence of multiple non-complementary strands is a situation for which standard short-read aligners are generally unequipped.

For these reasons, a number of bisulfite aligners have recently been created, the most popular of which is Bismark [2, 3] (see [4] for a general comparison of BS-seq aligners). Bismark, like BSmooth [5] and BS-Seeker2 [6], exploits the short-read aligners bowtie or bowtie2 [7] to perform alignments. While these aligners generally produce good results, they are quite slow, due to running up to 4 instances of bowtie simultaneously on the same computer as well as other implementation issues. Bismark and BS-Seeker2, in particular, have a higher than necessary false-positive rate due to not producing alignments with associated mapping qualities (MAPQ scores), which could otherwise be used to filter out methylation calls from alignments with too high a probability of being wrong, and how they decide which alignment to report. Similarly, while BSmooth produces alignments with MAPQ scores, they are based only on alignment to a single strand, so a read aligning equally well to multiple strands will have an aberrantly high MAPQ score. As an example, given a read that aligns with a MAPQ score of 42 to one strand and 40 to another, BSmooth will output the alignment with a MAPQ score of 42 and leave that MAPQ score unchanged. Had this MAPQ score instead been calculated in a context within which the alternative (MAPQ score of 40) alignment was known, the resulting MAPQ score would have appropriately been much lower. BSMAP [8], an older aligner, has previously been reported to require a similar amount of time as bismark to run but, because it uses its own aligner, produces somewhat better coverage [4]. Other recent bisulfite aligners, e.g. GNUMAP-bs [9], purport to have higher speed and accuracy at the expense of significantly increased memory requirements. In practice, though, the speed increase is marginal and accuracy increases are non-existent. As the alignment step is simply an intermediate between receiving raw data and actual statistical analysis, this creates an unnecessary experimental impediment.

To combat these issues, we have developed a novel bisulfite aligner, Bison (BISulfite alignment On Nodes of a cluster), written in C that exploits the increasing prevalence of computer-clusters to rapidly align BS-seq reads. Bison can utilize either 3 or 5 nodes, a master node to process alignments and one worker node per DNA strand for aligning reads. We also provide a second version, bison_herd, which scales to run on a semi-arbitrary number of cluster nodes, rather than being limited to 3–5 nodes, depending on library type. Like Bismark, BSmooth and BS-Seeker2, Bison utilizes bowtie2 to perform actual alignments, but produces alignments with recalculated MAPQ scores (a more general feature comparison is shown in Table 1). Because Bison can simultaneously use multiple nodes it requires less memory per-node. Furthermore, as the algorithm used by Bison to judge the best alignment is different from that used by the other aligners, its false-positive rate is decreased to that of bowtie2. Even when using identical resources, Bison performs its alignments in a fraction of the time required by all of the other compared aligners, except BSMAP. Bison produces more accurate results than all of the other compared aligners on simulated datasets. Unlike most other BS-seq aligners, bison_herd does not create temporary files, decreasing both I/O and space requirements. To easily facilitate statistical analysis, Bison comes with methylation extraction and conversion programs. Bison also provides the facility for easy quality control of aligned data and it can compute methylation metrics dependent upon these quality control results. Finally, bison is accompanied by a helper program that, unlike similar programs, uses both alignment position and methylation calls to mark likely PCR duplicates, which are then ignored during the methylation extraction and quality control process.

**Table 1:** **Feature comparison of the various compared aligners**

	Bismark	Bison/bison_herd	BSMAP	BSmooth	BS-Seeker2	GNUMAP-bs
Intermediate files	Yes	Yes/No	No	Yes	Yes	Yes
Compressed input	Yes	Yes	Yes	Yes	Yes	No
MPI	No	Yes	No	No	No	Yes
MAPQ scores	No	Yes	No	Yes	No	Yes
Creates M-bias plots	Yes	Yes	No	Yes	No	No
Per-read M-bias	Yes	Yes	No	No	No	No
Per-strand M-bias	No	Yes	No	No	No	No
Filter by MAPQ	No	Yes	No	Yes	No	No
Filter by Phred	No	Yes	No	Yes	No	No
Filter by M-bias	Yes	Yes	No	Yes	No	No
Mark duplicates	Yes	Yes	No	No	No	No
Methylation file format	bedGraph	bedGraph	custom TSV	custom TSV	Wig, custom TSV	custom TSV

## Implementation

**Figure 1:**
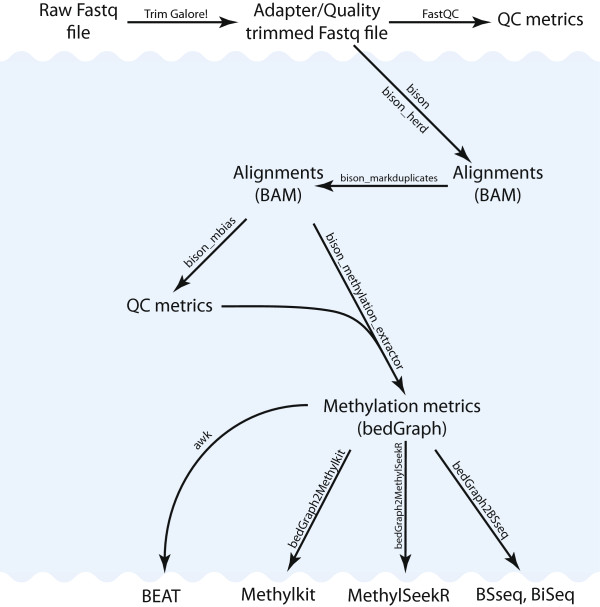
**Standard bisulfite-seq processing workflow.** Programs and steps for which Bison provides solutions are on a blue background.

The general workflow is depicted in Figure 1, with recommended steps external to Bison/bison_herd noted. As with other aligners, an index of the genome is created to facilitate faster mapping. This step needs to be performed only once. Two temporary copies of the genome are created, one C- > T and the other G- > A *in silico* converted. Prior to alignment, low quality bases and incorporated adapter sequences should be removed, as previously recommended [3]. We recommend Trim Galore! ( http://www.bioinformatics.babraham.ac.uk/projects/trim_galore/), as it is tailored toward bisulfite-converted reads and supports paired-end reads. An alternative to trimming is using local alignment. While Bison is capable of producing local alignments, the likelihood of incorrect methylation calls due to partial adapter alignment has caused us to default to producing end-to-end alignments. For the same reason, the comparisons included below contain only trimmed datasets.

Both Bison and bison_herd accept the resulting trimmed fastq files, which may also be compressed to save space. Bison_herd can also process a list of single or paired-end files, writing results to individual files. This is particularly convenient when processing multiple samples, as the genome and its indices only need to be read from disk and loaded into memory once. Bison *in silico* converts these reads on the master node as appropriate and writes the results to a file or files. The number and nature of the conversion is dependent upon whether the libraries were directional or non-directional and run single or paired-end. These converted reads are then aligned as appropriate by bowtie2 on the worker nodes. In bison_herd, the process is similar, except reads are sent unconverted via MPI from the master node to the worker nodes, where they are converted prior to being given via named-pipes to bowtie2.

As each worker node creates alignments, they are sent via MPI to the master node. There, the alignments are collated and a best alignment is selected (process described below). Subsequently, the methylation state of each base in each read is determined. If a read contains a mismatch, the methylation state of that base is set to ‘.’, to denote "not applicable". The results are then written to a BAM file using the samtools C API [10]. Unmapped reads may optionally be written to fastq files.

After performing quality control (see below), methylation metrics are normally extracted and written in bedGraph format. For paired-end reads, overlapping portions of each pair are processed as a unit so to not double-count methylation calls. It sometimes occurs that reads in a pair disagree on a methylation call, either because one read contains an N at a given position, or because both reads simply contain different high quality base calls. In the former case, Bison will always use the methylation call of the read not containing an N at the position in question. In the latter case, neither methylation call is used. The resulting methylation metrics are written for every C in a CpG, CHG, or CHH context to separate bedGraph files. A helper program is also included to merge metrics from both C’s in a CpG context. Other auxiliary scripts are provided to produce various metrics (e.g. per-CpG coverage), merge bedGraph files from technical replicates, and convert files for easy input into R for processing by methylKit [11], BSseq [5], BiSeq [12], BEAT [13] and MethylSeekR [14].

### Determining the best alignment and MAPQ recalculation

Like Bismark, Bison uses the alignment score (the auxiliary AS tag) of each alignment from each strand to determine the correct alignment to output. As mentioned above, if multiple strands produce equally good alignments for a read then that read is marked as being unmapped. This is in contrast to Bismark, which will declare the first alignment in its list as being best in these cases. For paired-end reads, the alignment scores are summed. When multiple valid alignments are found with one of them having a single highest score, the XS tag for that alignment is set to the highest alignment score of the other alignments. In cases where the best alignment already has a secondary alignment and that score is higher than those produced by alignment to other strands then the XS tag is unmodified. This modification of the XS tag allows for recalculation of the alignment mapping quality (MAPQ), which greatly increases the reliability of the resulting alignments (see below).

As in bowtie2, Bison calculates MAPQ scores using the aforementioned AS and XS values. As these values are set using alignments from all strands, the resulting MAPQ score will be more accurate than simply passing through the value calculated by bowtie2 (as is done by BSmooth). The actual algorithm used is a heuristic based on the percentile score of an alignment’s AS score after normalizing for the minimum valid and maximum possible such score. The higher this percentile, the higher the resulting MAPQ score. The difference between the AS and XS values further alters this score, where a smaller difference results in a lower MAPQ score.

### Marking duplicates

As is also the case in SNP calling, the presence of PCR duplicates can vastly inflate the coverage of a given region, resulting in false-positive findings in downstream analyses. Because of this, Bison also comes with a helper program, bison_markduplicates, to mark likely PCR duplicates. Bison will consider two alignments to be PCR duplicates if they align to the same strand of the same chromosome/contig and have identical 5’ coordinates. Furthermore, the methylation calls of the reads or read pairs are also compared and must match. For this, only methylation calls resulting from bases with Phred scores of at least 5 are included, to decrease errors due to a low quality base in one read/pair. In cases where a methylation call is made in one read/pair and not the other (e.g., due to a low Phred score or presence of a base other than C or T), the reads are declared to not differ at that position. This method, then, is somewhat conservative, though less so than if methylation calls were not included in determining duplicates. As paired-end reads are considered as a unit, rather than each read being considered separately, a duplicate can only occur if another pair of reads has these identical properties. The read or pair with the highest sum of base-call Phred scores is kept unmarked (this is also the case for samtools rmdup and picard markduplicates ( http://picard.sourceforge.net)). Like picard markduplicates, bison_markduplicates also incorporates soft-clipped bases when determining the 5’ coordinate.

### Quality control

**Figure 2:**
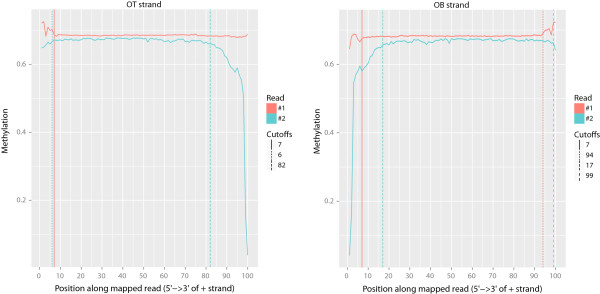
**M-Bias plots are useful for quality control.** The methylation level averaged per position in the reads should be nearly constant, but often shows bias at the 5′ and/or 3′ end of reads. The first (red) and second (blue) read in each pair is plotted separately. Likewise, results of reads mapping to the original top (OT, left) strand and original bottom (OB, right) are kept separate. When a non-directional library is used, graphs for the complementary to original top (CTOT) and complementary to original bottom (CTOB) are also produced. Vertical lines indicate the suggested bounds of the region for inclusion during methylation extraction.

Aside from adapter and quality trimming, the most important quality control step in BS-seq involves the creation of an M-bias plot (Figure 2), first introduced in [5]. While the reliability of the methylation calls should be constant across the length of the reads, there are often biases at both the 5’ and 3’ ends. This can be due to end-repair and decreasing read quality, among other reasons. For this reason, the methylated-C percentage of CpGs is calculated per read position and then plotted. As different strands may produce different biases, these metrics are calculated per strand. Likewise, the first and second read in each pair of paired-end reads often have different biases, so they are handled separately. The program used to compute M-bias metrics in Bison also performs filtering according to MAPQ and base Phred score in a manner identical to that in the methylation extractor.When bias, such as that seen in Figure 2, is found in a dataset, the methylation extractor that accompanies Bison can be instructed to ignore the affected portions of reads. As above, this ignored portion can depend on the strand to which a read aligns and, in the case of paired-end reads, on whether the read was the first or second in the pair. The program that produces the M-bias graphs prints suggested regions to ignore to the command line and labels the regions on the resulting graphs.

## Results and discussion

**Figure 3:**
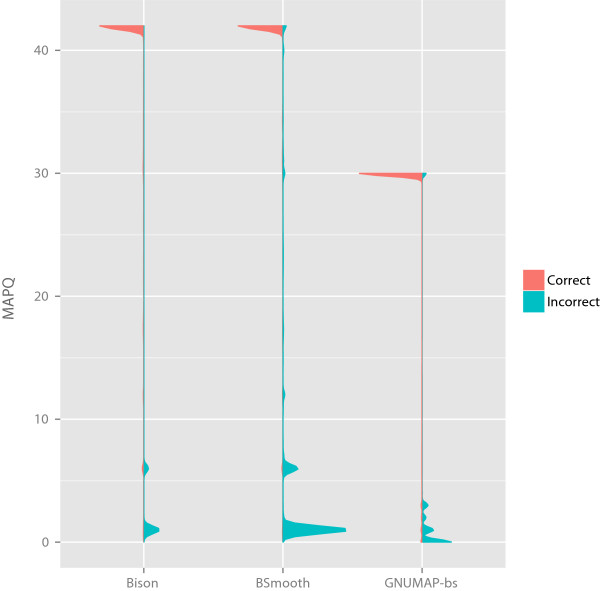
**Bison produces more accurate results than BSmooth or GNUMAP-bs.** The MAPQ density of correctly (red) and incorrectly (blue) aligned reads from the test dataset for alignments performed by Bison, BSmooth, and GNUMAP-bs. Bismark, BSMAP and BS-Seeker2 are not shown as they do not produce alignments with MAPQ scores. Scaling of the correct and incorrect metrics is performed separately, as the incorrect density would otherwise not be visible.

To compare the performance of Bison/bison_herd against Bismark (version 0.10.0), BSmooth (version 0.8.1), BSMAP (version 2.74), BS-Seeker2 (version 2.0.3) and GNUMAP-bs (GNUMAP version 3.0.2), we simulated 20 million 50 bp paired-end reads from a directional library with typical error profiles using Sherman ( http://www.bioinformatics.bbsrc.ac.uk/projects/sherman/). As these reads originated from known locations, we were able to assess not just the speed and resource requirements of each of the aligners, but also their accuracy. The results are presented in Tables 2 and 3 and Figure 3. As the output of bison_herd is identical to that of Bison, only Bison is included in Table 2. Using only reads that aligned with MAPQ scores of at least 10, as only these would be used for methylation calculations by default, Bison produced more correct and fewer incorrect alignments than Bismark. While BSmooth produced more correct alignments than Bison, it produced an equivalent increase in incorrect mappings, due to not recalculating MAPQ scores after determining to which strand each read (or pair, as is the case here) aligns. Other differences to BSmooth are due to Bison excluding from output reads that map equally well to multiple strands, as such alignments are highly inaccurate. Differences between the alignment accuracy of Bison and Bismark are due not only to recalculation of MAPQ scores by Bison, but also to incorporation of base quality scores in the calculation of alignment scores and Bismark’s more conservative threshold for calling an alignment valid. BSMAP produced a similar percentage of correct alignments to Bison, but had higher numbers of incorrect alignments even after filtering non-uniquely aligned reads. BS-Seeker2 performed similarly to BSMAP when only uniquely aligned reads were output. While GNUMAP-bs purports to have higher accuracy than Bismark or BSmooth, this is an artifact of it producing multiple alignments for each read. If a read produces multiple equally good alignments, only the first of these is counted here, since including all of them, as was presumably done by the GNUMAP-bs authors, would produce meaningless metrics (e.g., an aligner could always produce perfectly correct alignments by simply outputting all conceivable alignments). A violin plot of the correct and incorrect mappings produced by each program as a function of MAPQ is show in Figure 3, with the correct and incorrect metrics scaled independently. Table 3 illustrates how both Bison and bison_herd have much higher performance than any of the other tested aligners, except for BSMAP, even when limited to the same hardware and using the same version of bowtie2 with similar settings and identical numbers of threads. While earlier versions of BSMAP had a similar alignment rate to Bismark, more recent versions seem vastly faster than any of the aligners when using the same hardware, though there is a notable commensurate decrease in accuracy.

**Table 2:** **Alignment comparison of Bison with a variety of other aligners on the simulated dataset**

Program	Correct	Incorrect	Discarded
Bismark	82.41%	0.44%	17.15%
Bison	90.73% (87.09%)	2.01% (0.02%)	7.25% (12.89%)
BSMAP	89.20% (88.05%)	10.59% (2.53%)	0.21% (9.43%)
BSmooth	90.73% (88.17%)	9.19% (1.12%)	0.07% (10.71%)
BS-Seeker2	88.55%	2.06%	9.39%
GNUMAP-bs	84.64% (79.43%)	3.78% (0.27%)	11.57% (20.29%)

Numbers in parentheses are metrics when only alignments with MAPQ > =10 are included, expect for BSMAP, where they indicate metrics using only uniquely aligned reads (using the "-r 0" option).

**Table 3:** **Time requirement for each program with a variable number of available nodes to align the simulated dataset**

Program	Nodes (cores)	Time required (hh:mm:ss)	Read pairs/core/sec.	Options
Bismark	1 (12)	05:05:32	90.91	-p 5 –bam
Bison	1 (12)	01:29:25	310.66	-p 5
Bison	3 (36)	00:40:21	229.47*	-p 11
bison_herd	1 (12)	01:35:43	290.21	-p 5
bison_herd	3 (36)	00:39:21	235.30*	-@ 4 –p 11
bison_herd	5 (60)	00:21:24	259.61*	-mp 2 -@ 4 -p 11
bison_herd	9 (108)	00:13:55	221.78*	-mp 2 -@ 4 -p 11
BSmooth	1 (12)	07:44:02	59.86	-p 5 –no-mixed –no-discordant –no-unpaired
BSMAP	1 (12)	00:15:24	1803.75	-p 12
BSMAP	1 (12)	00:14:40	1898.94	-p 12 –r 0
BS-Seeker2	1 (12)	04:38:07	99.88	–aligner = bowtie2 –bt2-p 6 –bt2–end-to-end
GNUMAP-bs	1 (12)	04:26:53	104.08	–lib_type wt1 –read_type dna –nt_conv bs –-num_threads 12 –skip_blat 1 –pileup 1 –m 22 –a 0.90

*All cores in the master node are used for the calculation, even though they will not all be in use. Values are generally ~290 read pairs per core per second if this is not done.

**Figure 4:**
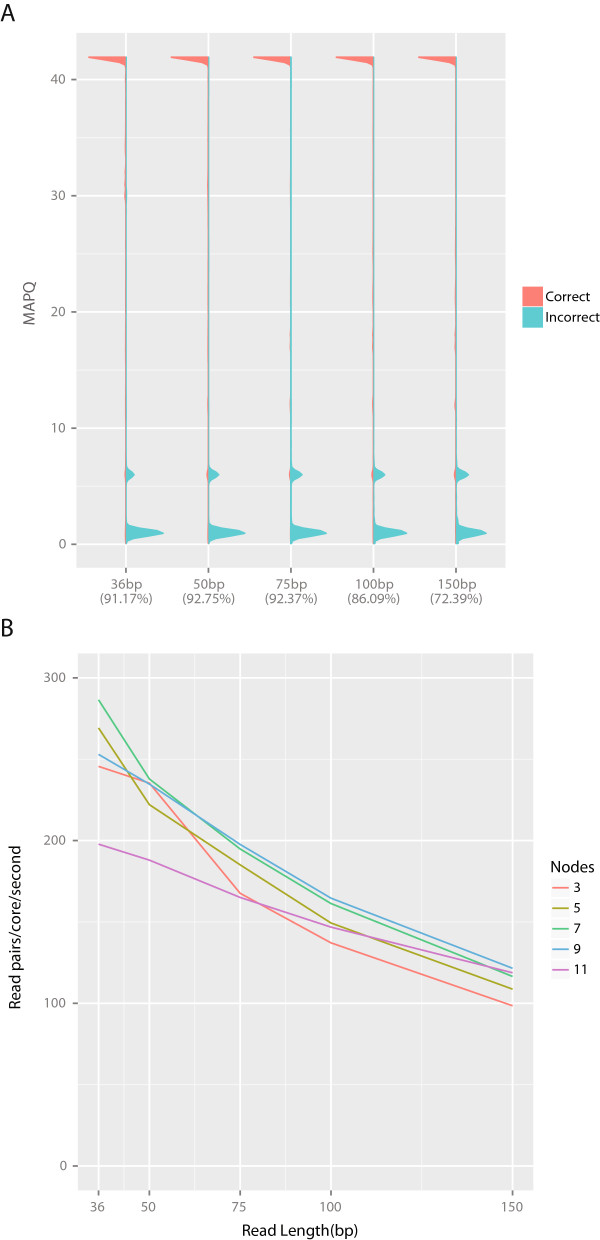
**Bison’s accuracy is consistent across read-length and its speed is generally consistent with increasing nodes.** Sherman was used to generate simulated directional datasets of variable length, as described above. The accuracy of the resulting alignments is consistent regardless of read-length, **A**. The percentages in parentheses under each read-length label are the total percentage of aligned reads (from 20 million original reads). The speed with which bison_herd produces alignments decreases with increasing read-length, **B**. This rate, however, is generally consistent when increasing numbers of cluster nodes are used. Note that in the test dataset, the MPI overhead required to use 11 nodes with shorter reads was sufficient to over-tax the MPI implementation and network. When this will occur will vary by cluster.

The accuracy of alignments produced by Bison and bison_herd are consistent with varying read lengths, Figure 4A and Table 4. It should be noted that the number of errors per read in these simulated datasets increases with read length, explaining the increase in discarded reads in the 100 and 150 bp datasets. The time required for alignments scales linearly with read length and is fairly stable with increasing numbers of available nodes, though this can become limited by the underlying MPI implementation and network architecture of the cluster, Figure 4B.

**Table 4:** **Bison produces consistently accurate alignments across read lengths**

Read length	Correct	Incorrect	Discarded
36 base pairs	88.41% (85.97%)	2.76% (0.01%)	8.83% (13.94%)
50 base pairs	90.73% (87.09%)	2.01% (0.02%)	7.25% (12.89%)
75 base pairs	90.58% (87.24%)	1.80% (0.01%)	7.76% (12.75%)
100 base pairs	84.52% (81.33%)	1.56% (0.01%)	13.91% (18.66%)
150 base pairs	71.13% (68.41%)	1.26% (0.01%)	27.61% (31.58%)

Numbers in parentheses are metrics when only alignments with MAPQ > =10 are included.

**Figure 5:**
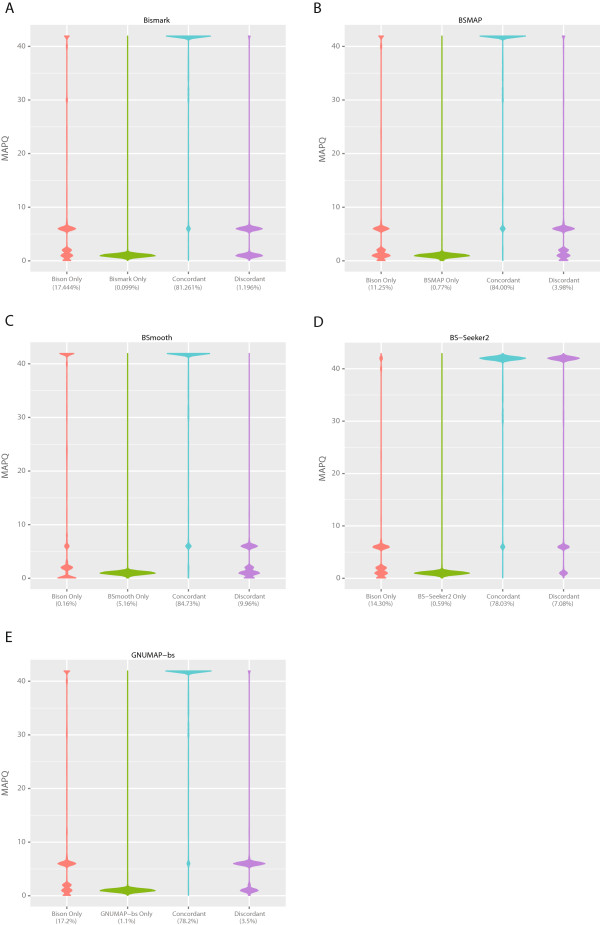
**MAPQ distribution of concordant, discordant, and unique alignments produced by Bison and the other compared aligners on the mouse WGBS dataset.** Reads from ERR192350 were aligned by both Bison and the other compared aligners (**A**, Bismark; **B**, BSMAP; **C**, BSmooth; **D**, BS-Seeker2; and **E**, GNUMAP-bs) and the resulting alignments compared according to the MAPQ score given to them by Bison. For alignments not reported by Bison, the MAPQ scores are what they would have been had Bison reported them. Alignments are considered concordant if both programs aligned them to the same position. Otherwise, they are considered discordant. Percentages below the labels are the percent of total mapped reads falling into each category.

**Figure 6:**
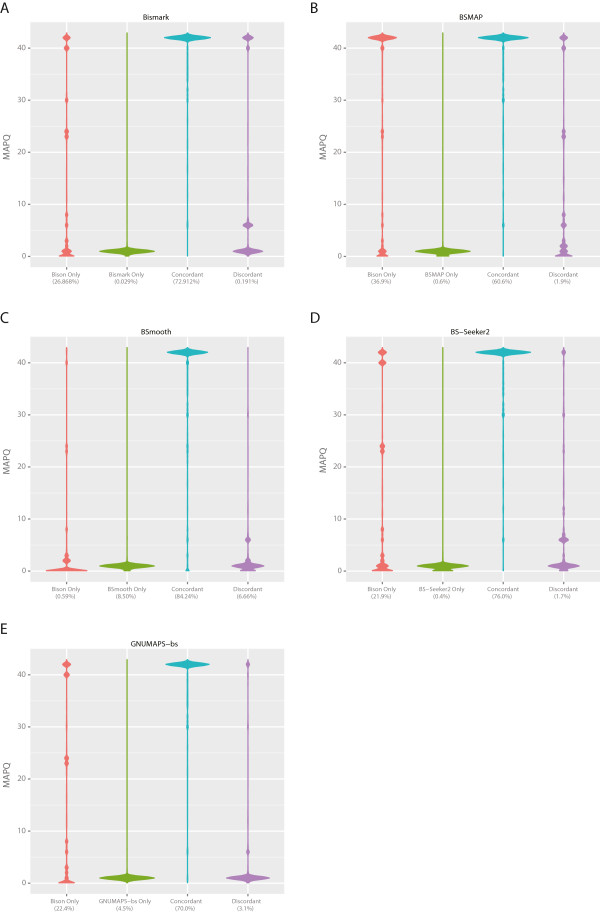
**MAPQ distribution on the human WGBS dataset.** Reads from SRR306438 were aligned by Bison and the other compared aligners (**A**, Bismark; **B**, BSMAP; **C**, BSmooth; **D**, BS-Seeker2; and **E**, GNUMAP-bs) and the resulting alignments compared as in Figure 5.

We compared the various aligners on three publicly available datasets. ERR192350 is a whole-genome bisulfite sequencing (WGBS) dataset using DNA from E6.5 mouse epiblasts and has 100 bp paired-end reads from a directional library [15]. SRR306438 is a human WGBS dataset using DNA from human sperm and has 100 bp paired-end reads from a directional library [16]. ERR034786 is a reduced-representation bisulfite sequencing (RRBS) dataset, using DNA from mouse sperm and has 40 bp paired-end reads from a non-directional library [17]. Each of these datasets was quality and adapter trimmed prior to alignment. For the whole-genome datasets, Bison had a higher alignment speed (measured in read pairs per CPU core per second) than the other aligners except for BSMAP, Tables 5 and 6. Further, Bison aligned a higher percentage of reads than all but BSmooth, however, the latter output inaccurate alignments that can originate from either strand. We looked at how concordant the alignments produced by Bison are to those produced by the other aligners. Here, a pair of alignments is termed concordant if both have the same start coordinate. As can be seen in Figures 5 and 6, the overwhelming majority of alignments are concordant between Bison and the other aligners. Discordant alignments are typically those Bison gives a low MAPQ, indicating that they would normally be ignored by it during methylation level calculation. As many of the other aligners do not produce alignments with MAPQ scores, they would include these likely incorrect alignments in this process. Alignments produced only by the other aligners would generally have very low MAPQ scores if they had been output by Bison, again due to mapping equally well to multiple strands.

**Table 5:** **Alignment comparison for the ERR192350 dataset**

Program	Nodes (Cores)	Time required (days + hh:mm:ss)	Read pairs/core/sec.	Percent aligned*
Bismark	1 (12)	2 + 14:12:56	90.95	76.42%
Bison	9 (108)	03:41:07	170.61	92.41% (72.46%)
BSMAP	1 (12)	04:13:04	1341.64	83.33%
BSmooth	1 (12)	6 + 06:10:42	37.68	97.79% (75.28%)
BS-Seeker2	1 (12)	2 + 21:10:15	81.81	80.03%
GNUMAP-bs	1 (12)	3 + 04:02:56	74.25	82.19% (74.66%)

*Numbers in parentheses are metrics when only reads with MAPQ > =10 are included.

**Table 6:** **Alignment comparison for the SRR306438 dataset**

Program	Nodes (Cores)	Time required (hh:mm:ss)	Read pairs/core/sec.	Percent aligned*
Bismark	1 (12)	03:17:30	129.94	50.72%
Bison	9 (108)	00:15:50	180.09	69.32% (57.11%)
BSMAP	1 (12)	00:55:52	459.36	62.33%
BSmooth	1 (12)	07:23:43	57.84	75.64% (59.71%)
BS-Seeker2	1 (12)	06:16:20	68.19	54.42%
GNUMAP-bs	1 (12)	13:43:30	31.16	58.54% (53.43%)

*Numbers in parentheses are metrics when only reads with MAPQ > =10 are included.

For the mouse WGBS dataset, we marked duplicates using bison_markduplicates and removed duplicates using Bismark’s deduplicate_bismark program to compare how Bison’s duplicate finding method compares to that of Bismark. Bismark found 55,860,458 pairs of duplicate alignments from the ERR192350 alignment produced by Bismark. Bison’s method marked 35,127,808 pairs of duplicate alignments from the same original file. The approximately 20 million fewer alignments marked as duplicates by Bison’s method are due to differences in methylation state within the reads.

**Figure 7:**
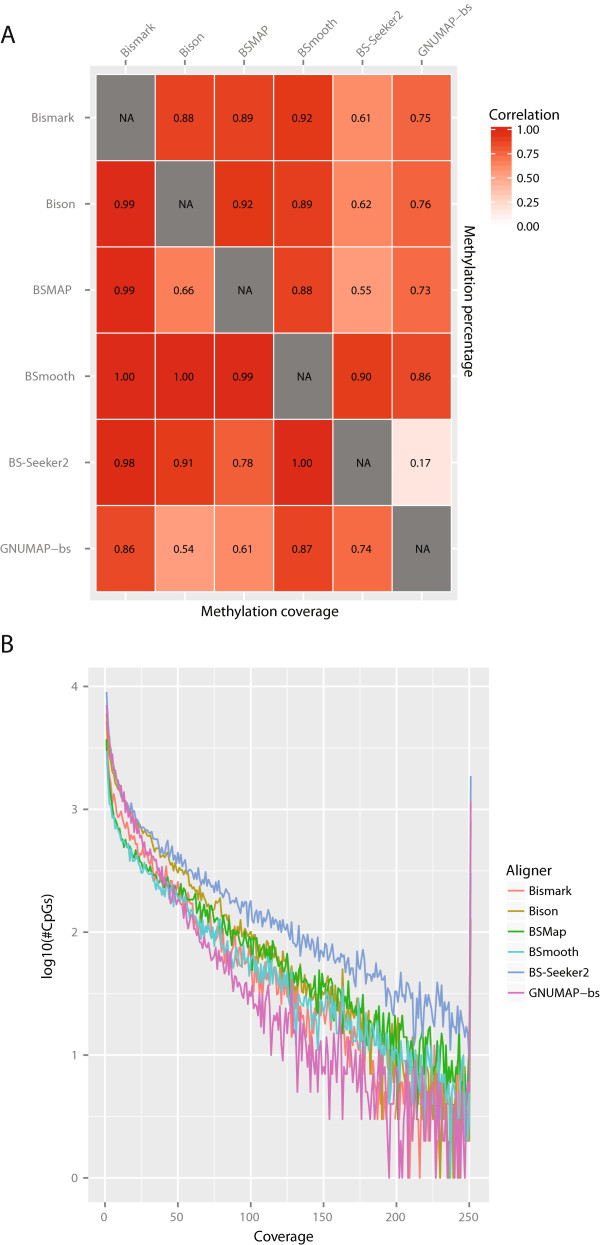
**Methylation percentage and coverage comparison using the RRBS dataset.** The Pearson’s correlation coefficients of the per-CpG estimated methylation percentages produced by each aligner are generally high, **A**, upper triangle. The correspondence between GNUMAP-bs and BS-Seeker2 and the other aligners, however, is notably lower, possibly due to their lower alignment rate. The Pearson’s correlation coefficients of the per-CpG coverage, **A**, lower triangle, is also generally high. The number of CpGs with a given coverage decreases rapidly as a function of coverage regardless of aligner, **B**. Note that CpGs with 251 or greater methylation calls are binned together. This binning produces the large spike at 251x coverage, which is likely due to PCR duplication.

For RRBS datasets, Bison, BSMAP and BS-Seeker2 offer the possibility to map to an *in silico* generated reduced representation genome. The time requirements, mapping rate and percentage alignment of the various aligners is presented in Table 7. For RRBS, a typical question is not just what the alignment rate is, but also how CpG coverage varies by aligner. As indicated in Table 1, Bison, Bismark and BSmooth can produce methylation-bias graphs and information from these was incorporated when calculating methylation levels from alignments produced by these aligners. Similarly, both Bison and BSmooth allow filtering methylation calls by both base Phred score and alignment MAPQ and both of those were utilized (the default for Bison is a minimum MAPQ score of 10 and a minimum Phred score of 5, so that was used for BSmooth as well). CpG coverage metrics are plotted in Figure 7 and shown in Table 8. For reference, there are 1,471,973 CpGs in the mm9 mouse reference genome sequence. Bison generally produces higher CpG coverage than the other aligners. BS-Seeker2 produces higher coverage than Bison, though this is likely due to it not filtering methylation calls by MAPQ and Phred score. It should be noted that GNUMAP-bs spreads fractional methylation calls arising from a single read across multiple positions, inflating its coverage metrics. The Pearson’s correlation of the methylation percentage and coverage produced by each aligner is shown in Figure 7. Only CpGs covered by both aligners are included in each comparison. The bowtie2-based aligners produce the most similar results, unsurprisingly. BS-Seeker2 and GNUMAP-bs are notable outliers, likely due to their low percentage alignment.

**Table 7:** **Alignment comparison with RRBS dataset ERR034786**

Program	Nodes (Cores)	Time required (hh:mm:ss)	Read pairs/core/sec.	Percent aligned*
Bismark	1 (12)	03:25:07	104.32	58.26%
Bison	9 (108)	00:14:32	163.60	71.59% (65.94%)
BSMAP	1 (12)	00:22:30	951.05	65.12%
BSmooth	1 (12)	07:12:01	49.53	97.40% (68.89%)
BS-Seeker2	1 (12)	02:50:40	125.38	32.21%
GNUMAP-bs	1 (12)	05:06:10	69.89	36.01% (27.74%)

*Numbers in parentheses are metrics when only reads with MAPQ > =10 are included.

**Table 8:** **Number of CpGs with a given coverage from the RRBS dataset**

Coverage	Bismark	Bison	BSMAP	BSmooth	BS-Seeker2	GNUMAP-bs
> = 1x	42,390	63,524	38,430	32,371	81,743	56,750
> = 3x	34,684	53,757	32,638	27,609	67,352	45,272
> = 5x	31,212	48,548	29,828	25,418	60,822	38,863
> = 10x	25,819	39,945	25,596	21,576	50,937	28,428
> = 30x	13,768	21,768	16,568	12,987	31,612	11,382
> = 50x	8,078	12,942	11,310	8,271	21,484	5,449
> = 100x	2,530	4,009	4,373	3,033	9,104	1,464

While Bison produces alignments more quickly than the other tested aligners, except for BSMAP, and had higher accuracy in the simulated dataset, there are still cases for which its use is less ideal. Bison does not handle color-space reads, which Bismark, for example, can handle easily. As Bison uses bowtie2 for alignment, its accuracy and speed are limited to that afforded by bowtie2. Furthermore, as Bison utilizes MPI and requires compilation, its setup is more complicated than aligners like Bismark or BSmooth, making it a less attractive option for small-scale or one-off experiments. Finally, while Bison can process targeted bisulfite sequencing datasets (e.g., those produced with Agilent’s SureSelect MethylSeq kits), the alignment process is not optimized to exploit the expected strand-bias of these datasets.

## Conclusions

Bison and bison_herd enable rapid and accurate alignment of BS-seq reads, such as those produced by the Illumina HiSeq. Both have equivalent to superior accuracy when compared to many previously published aligners. These programs also make performing quality control of the resulting mapped reads simple. As computer clusters are becoming increasingly prevalent in research settings, the speed of these tools removes the time-burden of alignment and simplifies the process of creating alignment pipelines, allowing the researcher to begin statistically analyzing experiments in a fraction of the time previously required. The open source nature of these programs and their usage of standard formats enable further adaptation and extension by end-users.

## Availability and requirements

**Project name:** Bison

**Project home page:**http://sourceforge.net/projects/dna-bison/

**Operating systems:** Linux, Mac OS

**Programming Languages:** C, python, R

**Other requirements:** GCC, bowtie2, MPI, Pthreads, Samtools

**License:** Modified MIT-style license, see LICENSE file for details

**Any restrictions to use by non-academics:** License required
